# Stromal Interaction Molecule 1 Promotes the Replication of vvIBDV by Mobilizing Ca^2+^ in the ER

**DOI:** 10.3390/v14071524

**Published:** 2022-07-13

**Authors:** Nana Yan, Yongqiang Wang, Zehua Chen, Aijing Liu, Yue Li, Bo Yang, Kai Li, Xiaole Qi, Yulong Gao, Li Gao, Changjun Liu, Yanping Zhang, Hongyu Cui, Qing Pan, Xiaomei Wang

**Affiliations:** 1 State Key Laboratory of Veterinary Biotechnology, Avian Immunosuppressive Diseases Division, Harbin Veterinary Research Institute, Chinese Academy of Agricultural Sciences, Harbin 150069, China; nnayan@126.com (N.Y.); czh1687@163.com (Z.C.); laj_91@126.com (A.L.); ly1996lion@163.com (Y.L.); yangbo2015hsy@163.com (B.Y.); likai01@caas.cn (K.L.); qixiaole@caas.cn (X.Q.); gaoyulong@caas.cn (Y.G.); gaoli@caas.cn (L.G.); liuchangjun@caas.cn (C.L.); zhangyanping03@caas.cn (Y.Z.); cuihongyu@caas.cn (H.C.); panqing@caas.cn (Q.P.); 2 Jiangsu Co-Innovation Center for the Prevention and Control of Important Animal Infectious Disease and Zoonose, Yangzhou University, Yangzhou 225009, China

**Keywords:** vvIBDV, STIM1, Ca^2+^

## Abstract

Infectious bursal disease virus (IBDV) is one of the main threats to the poultry industry worldwide. Very virulent IBDV (vvIBDV) is a fatal virus strain that causes heavy mortality in young chicken flocks. Ca^2+^ is one of the most universal and versatile signalling molecules and is involved in almost every aspect of cellular processes. Clinical examination showed that one of the characteristics of vvIBDV-infected chickens was severe metabolic disorders, and the chemical examination showed that their serum Ca^2+^ level decreased significantly. However, there are limited studies on how vvIBDV infection modulates the cellular Ca^2+^ level and the effect of Ca^2+^ level changes on vvIBDV replication. In our study, we found Ca^2+^ levels in the endoplasmic reticulum (ER) of vvIBDV-infected B cells were higher than that of mock-infected cells, and the expression level of stromal interaction molecule 1 (STIM1), an ER Ca^2+^ sensor, was significantly upregulated due to vvIBDV infection. The knock-down expression of STIM1 led to decreased Ca^2+^ level in the ER and suppressed vvIBDV replication, while the over-expressed STIM1 led to ER Ca^2+^ upregulation and promoted vvIBDV replication. We also showed that the inhibition of Ca^2+^-release-activated-Ca^2+^ (CRAC) channels could reduce vvIBDV infection by blocking Ca^2+^ from entering the ER. This study suggests a new mechanism that STIM1 promotes the replication of vvIBDV by mobilizing Ca^2+^ in the ER.

## 1. Introduction

Chickens and eggs are important sources of human food and are extensively consumed worldwide [1,2]. However, acute bursal disease (IBD) caused by vvIBDV causes heavy mortality (60–100%) in chickens, representing a considerable threat to the poultry industry [3]. Bursa of Fabricius (BF) is the main target of this virus, which targets B-lymphocytes, and hence the virus was named as infectious bursal disease virus (IBDV) [4,5]. Clinical examination revealed that IBDV-infected chickens had typical “spotted kidney” symptoms, indicating that their kidneys were severely damaged and suggesting that infected chickens might die of severe dehydration and electrolyte disturbance [6]. Previous scientists found that IBDV-infected chickens had serum uric acid (UA) concentrations above the normal comparison range [7]. As the kidney is the main organ of UA synthesis and excretion, and UA is the main end product of poultry metabolism [8], we hypothesized that although vvIBDV replicates in cells of BF, IBDV-infected chickens may die from severe metabolic disorders. However, studies on the relationship between vvIBDV infection and metabolism are limited.

In 1962, Cosgrove examined the chemical constituents of the blood of IBDV-affected chickens, and their most prominent finding was consistently low Ca^2+^ content of the serum [6]. Homeostasis is a mechanism to stabilize the cells by maintaining constant conditions [9,10,11]. Ca^2+^ homeostasis is pivotal for cells, reflecting the central importance of Ca^2+^ as one of the most universal and versatile signalling molecules that regulates almost all cellular processes such as metabolism, cell proliferation, and apoptosis [12,13,14]. Therefore, a network of Ca^2+^ transport and buffering systems has evolved to precisely control the cells [14]. As Ca^2+^ is so essential to living organisms, this study focuses on how vvIBDV affects Ca^2+^ levels of the host.

ER is the main internal Ca^2+^ store and plays an essential role in regulating Ca^2+^ homeostasis [15,16]. STIM1 is an ER Ca^2+^ sensor that responds to the loss of ER Ca^2+^ content and functions as a dynamic coordinator of cellular Ca^2+^ signals [17]. Orai1 is a Ca^2+^ channel in the plasma membrane (PM) [18]. STIM1 and Orai1 form the core components of CRAC channels [19], a unique and nearly ubiquitous class of channels that open in response to the loss of Ca^2+^ from the ER lumen and function as a main Ca^2+^ entry pathway into cells [20,21]. The decrease in Ca^2+^ levels in ER is sensed by the N-terminal arm of STIM1 [22]; this initiates a conformational change that promotes STIM1 oligomerization and localization to ER regions adjacent to the PM [23]. At PM, STIM1 interacts with Orai1 and activates CRAC channels, providing a pathway for sustained extracellular Ca^2+^ entry, known as store-operated Ca^2+^ entry (SOCE) [19]. In this process, STIM1 oligomerization is the critical transduction event, acting as a switch that triggers the self-organization and activation of STIM1-Orai1 clusters at ER-PM junctions [24].

In this study, we found vvIBDV infection increased the expression level of STIM1 and activated it. Activated STIM1 interacted with Orai1 and activates CRAC channels, thereby increasing the Ca^2+^ level in the ER and promoting vvIBDV infection, which could be abolished by the inhibition of CRAC channels.

## 2. Materials and Methods

### 2.1. Cells, Viruses, and Antibodies

DT40 (chicken B cell line) cells were maintained in RPMI-1640 Medium (R8758, Sigma, Burlington, MA, USA) supplemented with 10% foetal bowel serum (FBS), 2% chicken serum Sigma), 1% glutamine (25030-081, Gibco, Waltham, MA, USA), 50 μM 2-mercaptoethanol, and 1% penicillin-streptomycin in a 37 °C, 5% CO_2_ incubator. HEK293T human embryonic kidney cells were maintained in Dulbecco’s modified Eagle’s medium (DMEM) (c11995500BT, Gibco) supplemented with 10% foetal bowel serum (FBS), 1% glutamine, and 1% penicillin-streptomycin in a 37 °C, 5% CO_2_ incubator. Gx strain of vvIBDV was identified and preserved in our laboratory. Additionally, mouse monoclonal anti-IBDV (p)VP2 antibody was also produced and preserved in our laboratory. Antibodies used in the study include mouse anti-Flag M2 (F1804, Sigma, USA), rabbit anti-Flag (F2555, Sigma), rabbit anti-STIM1 antibody (produced by Abmart, Shanghai, China), rabbit anti-HA (H6908, Sigma), mouse-anti-HA (H9658, Sigma), mouse anti-β-actin monoclonal antibody (A1978, Sigma), rabbit anti-Myc (ab9106, Abcam, Cambridge, UK), goat anti-rabbit IgG H&L (Alexa Fluor 488) (A-11008, Invitrogen, Waltham, MA, USA), goat anti-mouse IgG H&L (Alexa Fluor 546) (A11003, Invitrogen), IRDye 680RD goat anti-mouse or goat anti-rabbit IgG H&L (LiCor Bio-Sciences, Lincoln, NE, USA), and IRDye 800CW goat anti-mouse or goat anti-rabbit IgG H&L (LiCor Bio-Sciences).

### 2.2. Construction of Plasmids

vvIBDV VP2 (1–441 aa), VP3 (756–1012 aa), VP4 (513–755 aa), and VP5 were cloned by inserting the viral genes of the vvIBDV Gx strain into the pCAGGS plasmid with an HA tag at the N-terminus. vvIBDV VP1 was constructed with an MYC tag at the C-terminal. All of these viral protein plasmids were constructed and preserved in our laboratory. STIM1 was cloned from the cDNA of DT40 cells using specific primers and was cloned into the pCAGGS plasmid with a Flag tag. Orai1 was cloned from the cDNA of DT40 cells using specific primers and was cloned into the pCAGGS plasmid with an MYC tag.

### 2.3. Reverse Transcription and Quantitative Real-Time PCR (qPCR)

Total RNA was extracted using a RNeasy Mini Kit (18274106, QIAGEN, Germantown, MD, USA), and 2 μg of RNA was reverse-transcribed into cDNA using a ReverTra Ace qPCR RT Master Mix with gDNA Remover (FSQ301, TOYOBO, Dublin, OH, USA) in a 10-μL reaction mixture. cDNA was analysed using qPCR by the Fluorescent Quantitative PCR Instrument (Mx 3005P, Aligent, Paradise, NV, USA). Specific primers and TaqMan probes for chicken 28s and IBDV VP5 were synthesized by Invitrogen (China). qPCR assays were performed with the following cycling conditions: 95 °C for 1 min for initial denaturation, followed by 40 cycles of 95 °C for 15 s for denaturation, 60 °C for 1 min, and collection of PCR product signals. All controls and infected samples were examined in triplicate on the same plate. cDNA quantities were normalized to 28S cDNA quantities measured from the same samples.

### 2.4. iTRAQ-Based Quantitative Proteomic Analysis

Primary chicken bursal B cells were infected with vvIBDV (MOI = 1) or mock infected, 2 h post infection, cells were collected and sent to LC-biotech (Zhejiang, China) to perform iTRAQ-based quantitative proteomic analysis. Briefly, protein samples were prepared by acetone precipitation method. The protein concentration was quantified using the Bradford method. Then, total protein (100 μg) was taken and digested with Trypsin Gold (Promega, Madison, WI, USA) with the ratio of protein: trypsin = 30:1 at 37 °C for 16 h. After digestion, peptides were dried and reconstituted in 0.5M TEAB and processed according to the manufacture’s protocol for 8-plex iTRAQ reagent (Applied Biosystems, Waltham, MA, USA). The iTRAQ-labelled peptide mixture was dissolved in 4 mL of buffer A (25 mM NaH2PO4 in 25% ACN, pH 2.7) and separated using an LC-20AB HPLC pump system (Shimadzu, Kyoto, Japan) with an Ultremex SCX column (4.6 × 250 mm, 5 μm, Phenomenex, Torrance, CA, USA). The analytical separation was performed using an LC-20AD nanoHPLC (Shimadzu, Kyoto, Japan) coupled to a triple TOF 5600 system (AB SCIEX, Concord, ON, Canada) fitted with a Nanospray III source (AB SCIEX, Framingham, MA, USA) and a pulled quartz tip as the emitter (New Objectives, Woburn, MA, USA). MS/MS data were matched using the Mascot search engine (Matrix Science, London, UK; version 2.3.02) against the NCBI-gallus (37,198 sequences) database (http://www.ncbi.nlm.nih.gov/data-hub/taxonomy/9031/, accessed on 10 February 2018) to identify the proteins. For protein quantitation, it was required that a protein contains at least two unique peptides. Quantitative protein ratios were weighted and normalized by the median ratio in Mascot. Ratios with *p*-values < 0.05, and only fold changes of >1.2 were considered as significant. Functional annotations of the proteins were conducted using Blast2GO program against the on-redundant protein database (NR; NCBI). The kegg database (http://www.genome.jp/kegg/, accessed on 13 February 2018) and the COG database (http://www.ncbi.nlm.nih.gov/COG/, accessed on 13 February 2018) were used to classify and group these identified proteins.

### 2.5. vvIBDV Infection and Titration

For viral infection, DT40 cells were counted by the cell counter and appropriately dilute viruses were incubated with cells for 4 h at a 41 °C, 5% CO_2_ incubator. Subsequently, viral inoculum was removed, and cells were maintained with 1640 complete medium until collection. The chicken embryos were used to titrate infectious progeny viruses after various treatments. Infected cell supernatants were harvested at specific time points after infection, and the titre of infectious viral progenies presented in the supernatants were determined in terms of ELD_50_/100μL using the Reed-Muench formula. All experiments were repeated three times, and the means and standard deviations were calculated.

### 2.6. Transfection, siRNA Knockdown, and Overexpression

DT40 cells were electroporated with Lonza cell line kit T (VVCA-1002, Lonza, Alps, Swiss) according to the manufacturer’s instructions. Three siRNAs specifically targeting the STIM1 mRNA of Gallus were designed by the Genechem Company (Shanghai, China) to study viral replication. siRNA sequences for knockdown of Gallus STIM1 included RNAi#1 (sense, 5′-CCAGGUUAGCGGUGAACAATT-3′), RNAi#2 (sense, 5′-GCAACACUCUGUUUGGAACTT-3′), RNAi#3 (sense, 5′-CCAUGCAAUCUCCUGCUUUTT-3′), and negative siRNA control (sense, 5′-UUCUCCGAACGUGUCACGUTT-3′). SiRNA transfections in 293T cells were performed using RNAiMAX (13778150, Invitrogen) according to the manufacturer’s instructions when cells were seeding. Flag-STIM1 transfections were performed at 24 h intervals. Then, 24 h after the second transfection, cells were harvested for further analysis. The siRNA with the highest knockdown efficiency was chosen for evaluating the influence of STIM1 on vvIBDV replication. For the replication study, siRNA-transfected cells were infected with the vvIBDV Gx strain at a multiplicity of infection (MOI) of 5 and cultured for an additional 48 h. Similarly, the cells of over-expression of STIM1 were infected with Gx strain at an MOI of 1 and cultured for an additional 72 h. Then, cell cultures and supernatants were collected to detect the yields of viral proteins and viral titres.

### 2.7. CO-IP and Western Blot Analysis

We performed CO-IP assays in both directions to confirm the interactions between vvIBDV and STIM1. First, HEK293T cells were seeded on 6-well plates and cultured for at least 12 h until cells were at 70% confluence before being transfected with pCAGGS-MYC-VP1, pCAGGS-HA-VP2, pCAGGS-HA-VP3, pCAGGS-HA-VP4, pCAGGS-HA-VP5, and/or pCAGGS-Flag-STIM1 by TransIT-X2^®^ Transfection System (Mirus, Madison, WI, USA). Next, 48 h after transfection, transfected cells were lysed in Cell lysis buffer for Western and IP (P0013, Beyotime, Shanghai, China). Then, supernatants were obtained by centrifuging and were incubated with 20 μL Anti-Flag M2 Affinity Gel (A2220, Sigma) at 4 °C for 6–8h or overnight. At the same time, we performed CO-IP assays in the opposite direction. The supernatants were incubated with 1 ug anti-HA rabbit mAb at 4 °C for 6–8h or overnight. After incubation with antibody, 25 μL Protein A/G-Agarose (A10001, Abmart) was added, and the samples were incubated at 4 °C overnight. Beads were washed five times with PBS, then boiled with 5×SDS loading buffer (P0015L, Beyotime) for 10 min. Subsequently, the samples were fractionated by electrophoresis on 12% SDS-polyacrylamide gels, and resolved proteins were transferred onto nitrocellulose membranes. After blocking with 5% skim milk, the membranes were incubated with rabbit anti-Flag and mouse anti-HA antibodies, followed by IRDye 800CW goat anti-mouse IgG secondary antibody and IRDye 680RD goat anti-rabbit IgG secondary antibody. The membrane blots were scanned using an Odyssey infrared imaging system.

### 2.8. Confocal Microscopy Assay

HEK293T cells were transfected with pCAGGS-Flag-STIM1 and/or pCAGGS-HA-VP2 for 24 h to observe the localization of VP2 and STIM1. At the same time, pCAGGS-Flag-STIM1 and/or pCAGGS-HA-VP4 were also transfected into HEK293T cells for 24 h to observe the localization of STIM1 and VP4. Then, the cells were washed with PBS three times and fixed with 4% formaldehyde for 30 min, followed by permeabilization with 0.1% Triton X-100 in PBS for 15 min. Samples were rinsed with PBS and blocked with 5% skim milk in PBS at 37 °C for 2 h before being incubated with mouse anti-HA (1:200) or/and rabbit anti-Flag (1:200) diluted in PBS for 2 h at 37 °C. Then, cells were washed three times with PBS and incubated with the secondary antibodies Alexa 488 anti-rabbit and Alexa 546 anti-mouse (1:500). Finally, cells were stained with 4′6-diamidino-2-phenylindole (DAPI) at room temperature for 15 min (C1005, Beyotime, China).

### 2.9. Airyscan Confocal Microscopy Assay

We performed the Airyscan confocal microscopy assay to improve the resolution and reveal more details about the intrinsic interaction between vvIBDV and CRAC channels. As described above, DT40 cells were infected at an MOI of 10 and were fixed at 24 h p.i., followed by permeabilization and block. Then, cells were incubated with mouse anti-VP2 mAb and rabbit anti-STIM1 antibody, followed by secondary antibodies and DAPI. After staining, the cells were washed 5 times and examined using a Zeiss confocal laser scanning microscopy with Airyscan (LSM800, Zeiss, Oberkochen, Germany). The images were fitting by Airyscan Processing.

### 2.10. Measurement of Cytosolic Free Ca^2+^ Concentration

Intracellular free Ca^2+^ concentration, [Ca^2+^]_i,_ was measured by using the fluorescent Ca^2+^ indicator fura-2/AM (F1225, Invitrogen) [25]. Briefly, 2.5 × 10^6^ cells were centrifuged at 500× *g*, 3 min, and washed twice with HBSS (24020117, Gibco), then incubated for 30 min at 37 °C away from light, next washed the cells twice with HBSS and then incubated for a further 30 min away from light to allow complete de-esterification of intracellular AM esters. Finally, cells were washed twice and suspended in Ca^2+-^free HBSS (14170112, Gibco) (CaCl_2_ was replaced by MgCl_2_) immediately prior to the measurement of fluorescence. Aliquots of cells were placed in a microwell plate, and fluorescence was measured in a Multiscan Spectrum (Enspire, PE) with the excitation wavelength being altered between 340 and 380 nm and emission fluorescence being recorded at 510 nm. All the measurements repeated 7 times and delayed 30s at each interval and all measurements were performed at room temperature. The fluorescence ratio R equals F340/F380, and F340 and F380 are the emission intensities at 340 and 380 nm excitation, respectively, corrected for autofluorescence. Fluorescence measured after the sequential addition of 0.1% Triton X-100 and then 50 mM ethylene glycol-bis (P-aminoethylether)-N,N,N′,N′-tetraacetic acid (EGTA) to the cell suspension provided the respective maximum fluorescence ratio (R_max_) and minimum fluorescence ratio (R_min_). [Ca^2+^]_i_ was calculated by the following equations:

[Ca^2+^]_I_ = Kd (R − R_min_)/(R_max_ − R) × b (1)

b = (F_380E_/F_380T_) (2)

where F_380T_ and F_380E_ are the emission fluorescence values at 380 nm excitation in the presence of Triton X-100 and EGTA, respectively. The equilibrium dissociation constant (Kd) for the Ca^2+^-fura-2 complex was 278 nM. Increases in fura-2 fluorescence indicated increased intracellular free calcium levels, because cellular cytosolic esterases cleave the membrane permeant ester group off the fura-2/AM derivative and leave the membrane-impermeant fura-2 trapped in the cytosol. The binding of Ca^2+^ shifts the absorbance spectra to shorter wavelengths (from 380 to 340 nm).

### 2.11. Measurement of Ca^2+^ Release from ER Ca^2+^ Stores

According to [26], thapsigargin (TG) (a specific inhibitor of the ER Ca^2+^-ATPase pump) (B6614, APExBio, Houston, TX, USA) was used to release Ca^2+^ from ER stores. A Ca^2+^-ATPase pump in ER membrane transports Ca^2+^ from the cytoplasm back into ER. When the ATPase pump in the ER is inhibited by TG, Ca^2+^ that leaks from the ER is not re-sequestered by the pumps, and Ca^2+^ accumulates in the cytosol. The TG-induced Ca^2+^ increase in cytosol is thought to represent Ca^2+^ released from the ER stores [26,27]. Briefly, fura-2/AM-loaded DT40 cells were washed twice in Ca^2+^-free HBSS and suspended in Ca^2+^-free HBSS. Before fluorescence measurement, 1 mM (final concentration) TG (B6614, APExBIO, USA) was added to the Ca^2+^-free HBSS immediately, and the [Ca^2+^]_i_ was measured as described above.

### 2.12. Measurement of Plasmalemma Permeability

According to [26], Ba^2+^ influx was used to measure plasmalemma permeability in vvIBDV infected DT40 cells, and 2-APB treated DT40 cells. Ba^2+^ is a Ca^2+^ surrogate that is transported across the cytoplasmic membrane through Ca^2+^ channels but is not a substrate for the carriers and pumps that normally transport Ca^2+^ across cell membranes [26]. However, Ba^2+^ will bind to fura-2 and change fluorescence in a fashion analogous to Ca^2+^ [28]. Briefly, fura-2/AM-loaded DT40 cells were suspended in Ca^2+^-free HBSS immediately before measurements were made. Then, 10 mM Ba^2+^ (final concentration) was added to the cells 2 min later. The fluorescence was measured at excitation wavelengths of 350 and 390 nm. An increased Ba^2+^ influx was indicated by an increase in the 350/390nm fluorescence ratio.

### 2.13. Cytotoxicity Test

Synta66 (HY-111325, MedCheExpress, Monmouth Junction, NJ, USA) was freshly prepared from stock solutions in DMSO. First, DT40 cells were pre-treated with Synta66 for a period of time at specific concentrations, then 1.5 × 10^6^ cells were resuspended with 1640 complete medium. Inoculated cell suspension (100 μL/well) in a 96-well plate (repeated each sample 3 times), and added 10 μL CCK-8 (CK04, DOJINDO) solution to each well of the plate. Next, incubated the plate for 2 h at 37 °C away from light. Finally, measured the absorbance at 450 nm using a microplate reader (BioTek, Winooski, VT, USA, ELx808).

### 2.14. Authentic Virus Infection of Synta66 Treated DT40 Cells

DT40 cells were infected with Gx strain of vvIBDV at an MOI of 5 for 4 h at 41 °C, 5% CO_2_ to let viruses enter the cells; then, the virus inoculum was removed. Synta66 was diluted in DMSO and added to cells at indicated concentrations. An equivalent percentage of DMSO was used as vehicle control. Cells were then incubated at a 41 °C, 5% CO_2_ incubator for 48 h.

### 2.15. Animals and Ethics Statement

The specific-pathogen-free (SPF) chickens were purchased from the State Resource Center of Laboratory Animal for Poultry (Harbin, China). This study was carried out in strict accordance with the recommendations in the Guide for the Care and Use of Laboratory Animals of the Ministry of Science and Technology of China. The use of SPF chickens and animal experiments were approved by the Animal Ethics Committee of Harbin Veterinary Research Institute of the Chinese Academy of Agricultural Sciences and performed in accordance with the animal ethics guidelines and approved protocols (SYXK (Hei) 2017-009).

### 2.16. Animal Experiments

3-week-old SPF chickens were randomly divided into two groups, the mock group (*n* = 19) and the vvIBDV challenged group (*n* = 19). The mock group was received 200 μL PBS via the ocular and intranasal routes as the negative control; the challenged group was infected with 10^3^ ELD_50_ (200 μL) of Gx strain of vvIBDV via the same route. From 1 to 3 d p.i., three chickens randomly selected each day from all groups were euthanized for necropsy and examination of pathological changes. The remaining chickens in each group were used to calculate the survival rate. Some parts of bursae at 3 d p.i. were fixed immediately in 10% neutral buffered formalin and were stained with haematoxylin and eosin for further histopathological, and some parts at 2 d p.i. were used for electron microscope examination. Serum was collected to test Ca^2+^ concentration, which was performed in Third Affiliated Hospital, Heilongjiang University of Chinese Medicine.

### 2.17. Statistical Analysis

Statistical analysis was performed with the unpaired *t*-test. *p* values of less than 0.05 were considered statistically significant. Data are reported as means ± standard deviations (SD).

## 3. Results

### 3.1. vvIBDV-Infection Induces A Significant Reduction of Serum Ca^2+^ Levels

**Figure 1:**
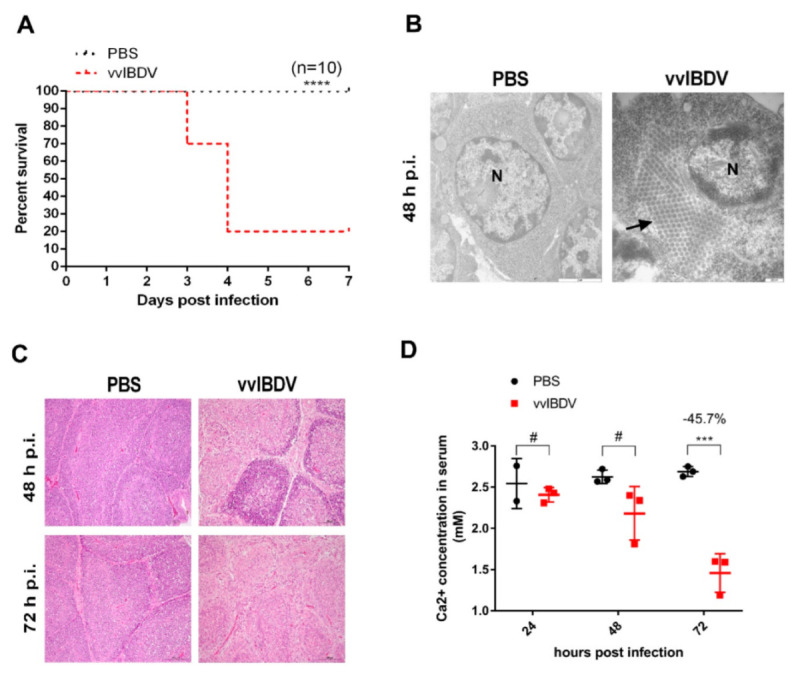
vvIBDV induces significant reduction of serum Ca^2+^ levels. Two-week-old SPF chickens were randomly divided into 2 groups (*n* = 38), mock group (*n* = 19), vvIBDV challenged group (*n* = 19). The mock group received 200 uL PBS via the ocular and intranasal routes as negative controls, and the challenged group were infected with 10^3^ ELD_50_ (200 uL) of Gx strain of vvIBDV via the same process. (**A**) Seven-day percent survival of mock infected chickens and vvIBDV challenged groups (*n* = 10). (**B**) Electron microscope examination of the bursal sections at 48 h p.i. The black arrow indicated the virions. N = nucleus. The scale bar showed at bottom of right in each individual picture. (**C**) The histopathological examination of the bursal sections (haematoxylin and eosin staining) at 48 and 72 h p.i., respectively. Scale bars, 100 μm. (**D**) Serum Ca^2+^ concentration of mock-infected chickens and vvIBDV-challenged chickens. *** *p* < 0.001; **** *p* < 0.0001; #, no significance.

Through pathogenicity experiments, we found that the seven-day survival rate of vvIBDV-challenged chickens was only 20% (Figure 1A). Electron microscopy showed that a large number of viruses accumulated in the bursa on the second day post-infection (d p.i.) (Figure 1B). Histology results showed a severe decrease in the B lymphocytes at 2–3 d p.i. (Figure 1C). These results showed that vvIBDV infection leads to high mortality and severe bursa lesion in chickens. We tested the concentration of Ca^2+^ in the serum and found the Ca^2+^ concentration of infected chicken decreased by 45.72% at 3 d p.i. (Figure 1D). This result suggested that chickens that died as a result of vvIBDV infection experienced severe Ca^2+^ disturbance, which might be an important factor of their death.

### 3.2. vvIBDV-Infection Increases Ca^2+^ Levels in the ER

**Figure 2:**
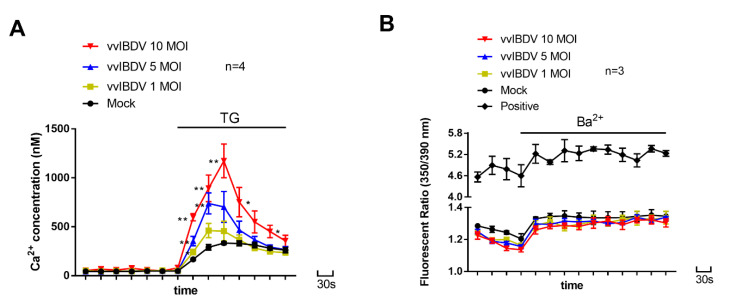
vvIBDV increases Ca^2+^ levels in the ER of infected cells. (**A**) Intracellular Ca^2+^ concentration was measured in fura-2/AM-loaded DT40 cells suspended in Ca^2+^-free HBSS. Cells were mock-infected or infected at MOI of 1, 5 or 10, and the cells were harvested and examined at 24 h p.i. In the absence of TG, the results were cytosolic Ca^2+^ levels. When TG was added to fura-2/AM-loaded cells to a final concentration of 1 μM, the fluorescence measurements were believed to represent the Ca^2+^ levels of ER stores (*n* = 4). Each unit represents 30 s. (**B**) Ba^2+^ influx in mock-infected and vvIBDV-infected DT40 cells. Cells were harvested at 24 h p.i. Fura-2/AM-loaded cells were incubated in Ca^2+^-free HBSS before measurements were made. Ba^2+^ at a final concentration of 10 mM was added to the cells 2 min later (*n* = 3). Each unit represents 30 s. Mean±SD were calculated based on three or four independent experiments. * *p* < 0.05; ** *p* < 0.01.

To detect whether Ca^2+^ concentration in vvIBDV-infected cells has changed, we analysed intracellular Ca^2+^ levels of vvIBDV-infected DT40 cells at 24 h post-infection (h p.i.). Our results showed that Ca^2+^ levels in the cytoplasm remained stable, while Ca^2+^ levels in the ER increased in a dose-dependent manner in vvIBDV-infected cells (Figure 2A). We also measured cell viability at a MOI of 10 at 24 h post-infection. Results showed that there was no significant difference in cell viability between infected cells and mock-infected cells (data not shown). These results demonstrate that vvIBDV infection leads to an increase in Ca^2+^ levels in the ER, but not in the cytoplasm.

To rule out the cell membrane permeability change as the cause of intracellular Ca^2+^ levels’ modulations, we examined basal Ba^2+^ influx in vvIBDV-infected cells (at 24 h p.i.) to detect cell membrane permeability. As a positive control for measuring changes in the membrane permeability, 0.02% Triton X-100 was used. Our results showed that, in mock and infected groups, the addition of Ba^2+^ to cells incubated in Ca^2+^-free HBSS buffer led to an insignificant increase in the fluorescence ratio, and their Ba^2+^ influx was much lower than the positive control. These results suggest that the cell membrane permeability does not differ between vvIBDV-infected cells and mock-infected cells (*p* > 0.05), and changes in cell membrane permeability are not responsible for increased ER Ca^2+^ levels observed in vvIBDV-infected cells.

### 3.3. Endogenous STIM1 Expression Is Upregulated by vvIBDV Infection

**Figure 3:**
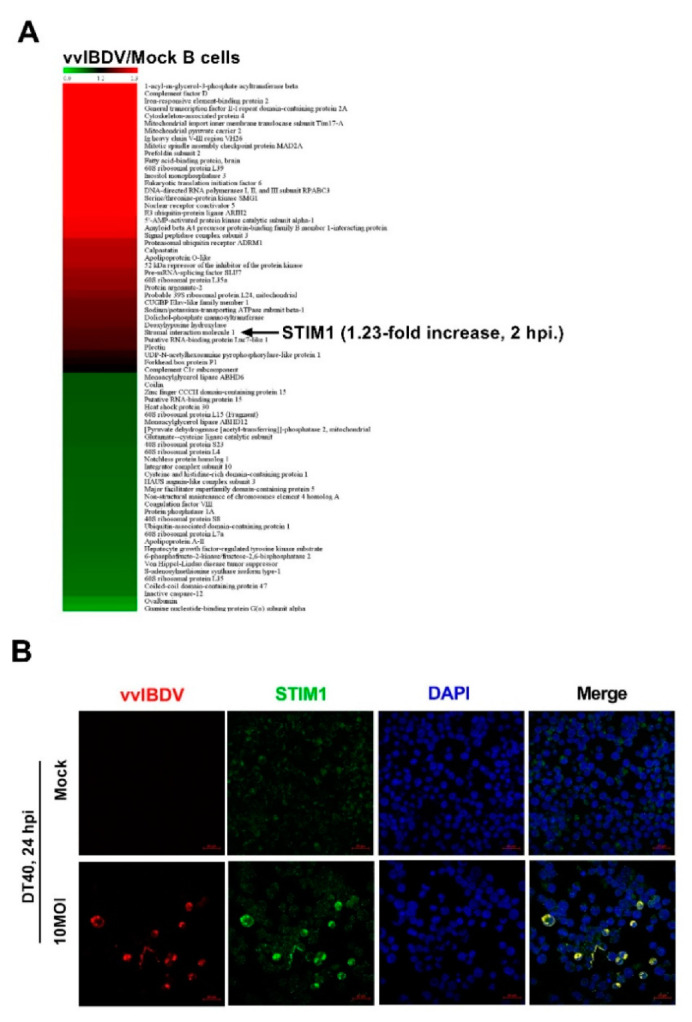
Endogenous STIM1 expression is upregulated by vvIBDV infection. (**A**) iTRAQ was conducted on vvIBDV- and mock-infected B lymphocyte samples. The expression level of STIM1 was found to be higher in the vvIBDV-infected group than in the mock-infected group at 2 h p.i. (1.23-fold). (**B**) The fluorescence brightness of STIM1 in vvIBDV-infected cells was significantly higher compared with mock-infected cells. DT40 cells were infected with vvIBDV at a MOI of 10, cells were fixed at 24 h p.i. and processed for dual-labelling. Cell nuclei were counterstained with DAPI (blue), VP2(red) and STIM1 (green) proteins were visualised by immunostaining with mouse anti-VP2 antibody and rabbit anti-STIM1 antibody, respectively, and were analysed using confocal microscopy at the same laser intensity. Scale bars, 20 μm.

To identify potential host proteins associated with Ca^2+^, we performed iTRAQ labelling of differentially expressed proteins in vvIBDV-infected and mock-infected B cells. Our results showed that viral infection led to overexpression of many host proteins, among which we found STIM1 to be of particular interest as STIM1 modulates ER Ca^2+^ levels directly (Figure 3A). The expression level of STIM1 was higher in vvIBDV-infected group than in the mock-infected group (1.23-fold increase) at 2 h p.i. STIM1 is an ER Ca^2+^ sensor, which plays a significant role in Ca^2+^ regulation [29]. To investigate the relationship between STIM1 expression level and vvIBDV infection, we used laser confocal microscopy for intuitive observation. Briefly, DT40 cells were infected with vvIBDV of Gx strain at a multiplicity of infection (MOI) of 10, and the cells were fixed at 24 h p.i. Fixed cells were incubated with mouse anti-VP2 antibody, rabbit anti-STIM1 antibody, and DAPI. It can be observed that the fluorescence intensity of STIM1 in infected cells was significantly higher than in mock-infected cells (Figure 3B). These results suggest that vvIBDV infection induces the upregulation of endogenous STIM1 protein levels.

### 3.4. STIM1 Knockdown-Mediated Reduction in ER Ca^2+^ Levels Impair vvIBDV Replication

**Figure 4:**
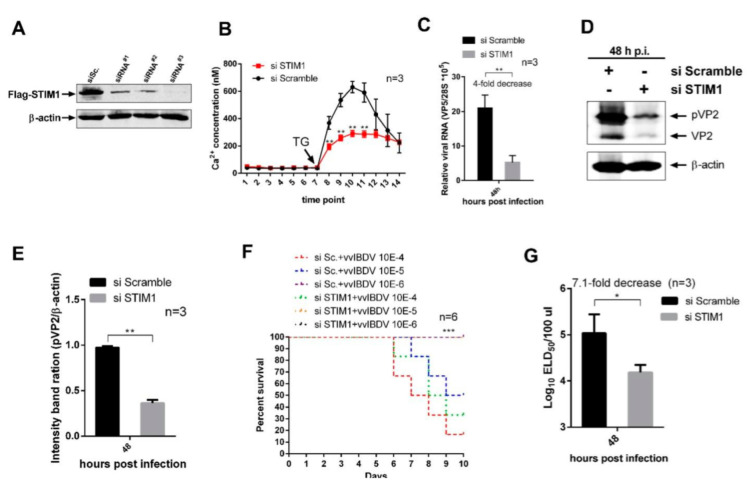
STIM1 knockdown-mediated reduction in ER Ca^2+^ levels impair vvIBDV replication. (**A**) Effects of STIM1 RNAi on the expression of exogenously gallus STIM1. HEK293T cells were transfected with siRNA (RNAi#1, RNAi#2, or RNAi#3) or scrambled siRNA. Cell lysates were harvested at 24 h after the secondary transfection of STIM1 expression plasmid and examined by WB with anti-FLAG antibody. Β-actin expression was used as a protein loading control. (**B**) Scrambled RNAi cells and STIM1 RNAi cells were infected with vvIBDV as a MOI of 5. Cells were harvested 24 h p.i. and intracellular Ca^2+^ concentration was measured as described in Figure 2A (*n* = 3). Each unit represents 30 s. (**C**) Scrambled RNAi cells and STIM1 RNAi cells were infected with vvIBDV at a MOI of 5. Cells were harvested at 48 h p.i. and RNA levels of VP5 were determined by RT-qPCR (*n* = 3). (**D**) Scrambled RNAi cells and STIM1 RNAi cells were infected with vvIBDV as in B. Cells were harvested at 48 h p.i. and examined by WB with antibodies against pVP2 and VP2. Β-actin expression was used as an internal control. (**E**) Relative intensities of viral protein pVP2 in STIM1 RNAi-treated cells (*n* = 3). (**F**,**G**) Scrambled RNAi cells and STIM1 RNAi cells were infected as in B. At 48 h p.i., the infected cell supernatants were harvested and the chicken embryos were inoculated. The representative percent survival of the chicken embryos was recorded in ((**E**), *n* = 6) and the infectious viral loads in the supernatants were determined by ELD_50_ analysis ((**F**), *n* = 3). Mean ± SD were calculated based on three independent experiments. * *p* < 0.05; ** *p* < 0.01; *** *p* < 0.001.

To study the role of STIM1 in regulating the Ca^2+^ levels in the ER, we screened three specific siRNAs against STIM1 and found that one of them could effectively reduce STIM1 expression (Figure 4A). This siRNA was used to knock down STIM1 in DT40 cells, followed by infection with vvIBDV (MOI of 5). At 24 h p.i., intracellular Ca^2+^ levels were examined. We found no significant difference in cytosolic Ca^2+^ levels between STIM1-knockdown group and scramble control group. However, there was a significant decrease in Ca^2+^ levels in the ER of STIM1-knockdown group (Figure 4B). The effect of STIM1 knockdown on vvIBDV replication was studied. At 48 h p.i., Western blot (WB) results showed that expression levels of viral proteins pVP2 and VP2 were downregulated in STIM1-knockdown group (Figure 4C,D). We also found the relative expression of viral RNA transcript decreased by 4-fold in STIM1-knockdown group compared with that in scrambled siRNA control group (Figure 4E). Further, the virus titre was checked in the supernatants of vvIBDV-infected cells by using ELD50 assay. We observed that STIM1 knockdown resulted in a 7.1-fold decrease in extracellular infectious progeny virus titre (Figure 4F,G). These results showed that knockdown of STIM1 suppressed vvIBDV replication. In conclusion, our results demonstrate that the knockdown of STIM1 decreases Ca^2+^ levels in ER, which impairs vvIBDV replication.

### 3.5. STIM1 Overexpression-Mediated Increase in ER Ca^2+^ Levels Enhance vvIBDV Replication

**Figure 5:**
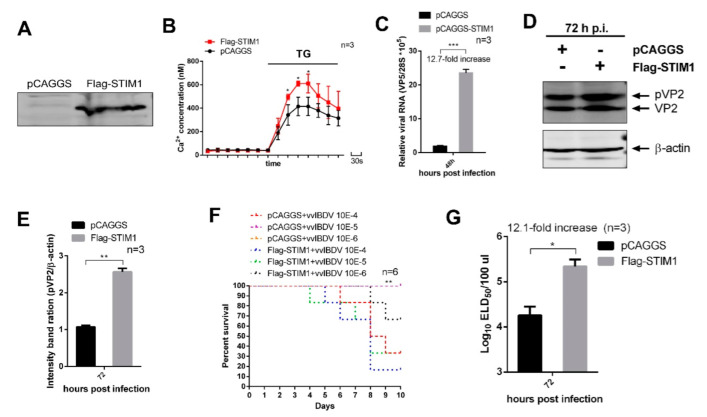
STIM1 overexpression-mediated increase in ER Ca^2+^ levels enhance vvIBDV replication. (**A**) DT40 cells were transfected with plasmids pCAGGS or pCAGGS-Flag-STIM1, 24 h later, results were observed by WB. (**B**) STIM1-overexpression cells and empty-vector cells were infected with vvIBDV at a MOI of 1. The cells were harvested at 24 h p.i. and the intracellular Ca^2+^ concentration was measured as described in Figure 2A. Each unit represents 30 s. (**C**) STIM1-overexpression cells and empty-vector cells were infected as (**B**). Cells were harvested at 72 h p.i. and RNA levels of VP5 were determined by RT-qPCR (*n* = 3). (**D**) STIM1-overexpression cells and empty vector cells were infected as (**B**). Cells were harvested at 72 h p.i. and examined by WB with antibodies against pVP2 and VP2. β-actin expression was used as an internal control. (**E**) Relative intensities of viral protein pVP2 in STIM1 overexpression cells (*n* = 3). (**F**,**G**) STIM1 overexpression cells and empty vector cells were infected with vvIBDV as (**B**). At 72 h p.i., the percent survival of the chicken embryos was recorded in ((**E**), *n* = 6) and the infectious viral loads in the supernatants ((**F**), *n* = 3) were determined as described in Figure 4F. Mean ± SD were calculated based on three independent experiments. * *p* < 0.05; ** *p* < 0.01; *** *p* < 0.001.

We also investigated the influence of overexpression of STIM1 on the ER Ca^2+^ levels and vvIBDV replication. DT40 cells were transfected with pCAGGS or pCAGGS-Flag-STIM1 plasmids, and the result of WB indicated that pCAGGS-Flag-STIM1 was successfully overexpressed in DT40 cells (Figure 5A). To study whether overexpression of STIM1 leads to increased Ca^2+^ levels in the ER, at 24 h p.i., intracellular Ca^2+^ levels were examined. We found no significant difference in cytosolic Ca^2+^ levels between STIM1-overexpression group and control group. However, there was a significant increase in ER Ca^2+^ levels in STIM1-overexpression cells (Figure 5B).

To study the effect of STIM1 overexpression on vvIBDV replication, 24 h after transfection, cells were infected with 1 MOI of vvIBDV. At 72 h p.i., we found that STIM1 overexpression upregulated the amount of viral RNA transcript by 12.7-fold compared with empty vector-transfected cells (Figure 5C). WB results showed that the expression levels of viral proteins pVP2 and VP2 were significantly upregulated in cells overexpressing STIM1 (Figure 5D,E). Further, we tested the titre of progeny viruses in supernatants of vvIBDV-infected cells by using the ELD50 assay. The results showed that overexpression of STIM1 resulted in a 12.1-fold increase in extracellular viral yields at 72 h p.i. (Figure 5F,G). These results showed that overexpression of STIM1 enhances vvIBDV replication. In conclusion, our results demonstrate that overexpression of STIM1 increases Ca^2+^ levels in ER, which promotes vvIBDV replication.

### 3.6. Viral Proteins VP2 and VP4 Interact with STIM1

**Figure 6:**
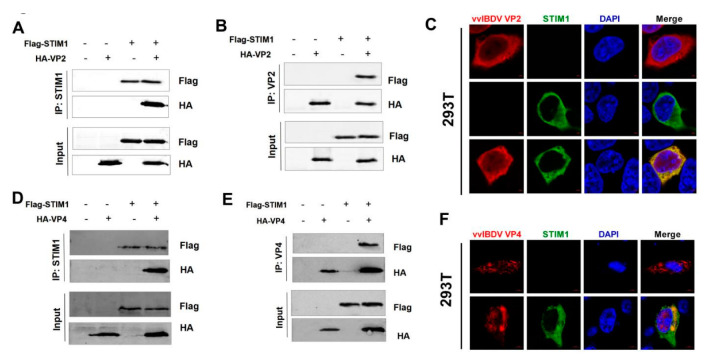
Viral proteins VP2 and VP4 interact with STIM1. (**A**) HEK293T cells were transfected with pCAGGS-HA-VP2 and pCAGGS-Flag-STIM1 expression plasmids. Cell lysates were prepared at 36 h p.i. and immunoprecipitated with anti-FLAG antibody and immunoblotted with anti-FLAG or anti-HA antibodies. (**B**) HEK293T cells were transfected as A. Cell lysates were prepared at 36 h p.i. and immunoprecipitated with anti-HA antibody and immunoblotted with anti-FLAG or anti-HA antibodies. (**C**) HEK293T cells were transfected with pCAGGS-HA-VP2 and/or pCAGGS-FLAG-STIM1 for 24 h and then fixed and processed for dual-labelling. Cell nuclei were counterstained with DAPI (blue), VP2 and STIM1 proteins were visualised by immunostaining with anti-HA and anti-FLAG antibodies (1:200), respectively, and were analysed using confocal microscopy. VP2 staining is shown in red, and STIM1 staining is shown in green; merged images are shown with areas of co-localisation in yellow. Scale bars, 2 μm. (**D**) HEK293T cells were transfected with pCAGGS-HA-VP4 and pCAGGS-FLAG-STIM1 expression plasmids. Cell lysates were prepared and immunoprecipitated as A. (**E**) HEK293T cells were transfected as C. Cell lysates were prepared and immunoprecipitated as B. (**F**) HEK293T cells were transfected with pCAGGS-HA-VP4 and/or pCAGGS-FLAG-STIM1 for 24 h and treated as E. VP4 staining is shown in red, and STIM1 staining is shown in green; merged images are shown with areas of co-localisation in yellow. Scale bars, 5 μm.

To further analyse the possible mechanisms underlying the influence of STIM1 on the progression of vvIBDV replication by searching for viral proteins that interact with STIM1. We transfected HEK293T cells with plasmids expressing pCAGGS-Flag-STIM1, pCAGGS-MYC-GxVP1, pCAGGS-HA-GxVP2 (1-441 aa), pCAGGS-HA-GxVP3 (756-1012 aa), pCAGGS-HA-GxVP4 (513-755 aa), and pCAGGS-HA-GxVP5, followed by co-immunoprecipitation (CO-IP) assays with anti-Flag antibody, and WB with anti-Flag, anti-MYC, or anti-HA monoclonal antibodies. Results showed that when lysates of cells expressing both HA-GxVP2 and Flag-STIM1 were immunoprecipitated with anti-Flag antibody, pCAGGS-HA-GxVP2 was detected in the precipitate. Similarly, in cells expressing both HA-GxVP4 and Flag-STIM1, the HA-GxVP4 was detected in the precipitate. These results indicated that both VP2 and VP4 interacted with STIM1 (Figure 6A,D). Other viral proteins had no interactions with STIM1 (data not shown). To confirm that the observed interactions were bi-directional, we performed CO-IP assays with HA antibody and WB with anti-Flag, or anti-HA monoclonal antibodies. Our results showed that Flag-STIM1 could be immunoprecipitated by both VP2 and VP4, indicating that STIM1 interacts with both viral proteins (Figure 6B,E).

To determine the subcellular localization of VP2/VP4 and STIM1, HEK293T cells were transfected to express HA-GxVP2/HA-GxVP4 and Flag-STIM1. After 24 h, cells were fixed and incubated with mouse anti-HA antibody, rabbit anti-Flag antibody, and DAPI, and analysed by confocal microscopy. We observed complete co-localization of VP2 with STIM1 (Figure 6C), whereas VP4 partially co-localized with STIM1 (Figure 6F).

### 3.7. vvIBDV-Infection Activates STIM1-Orai1 Dependent Ca^2+^ Pathway

**Figure 7:**
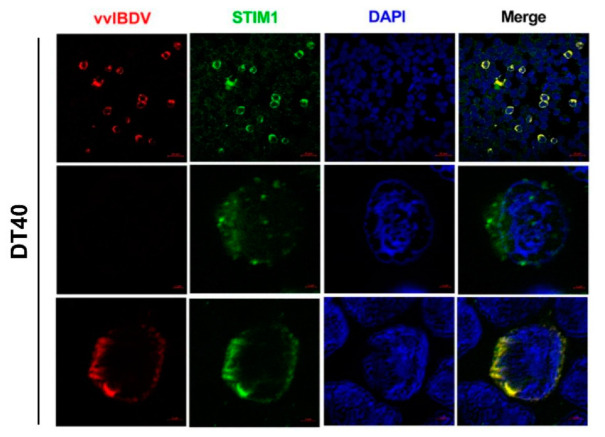
vvIBDV activates STIM1-Orai1 dependent Ca^2+^ pathway. 10MOI-infected DT40 cells were fixed and processed for duel-labelling at 24 h p.i. Cell nuclei were counterstained with DAPI (blue), VP2 and STIM1 proteins were visualised by immunostaining with anti-VP2 and anti-STIM1 antibodies, respectively, and were analysed using confocal microscopy. The two single cells chosen from this view were fitted by Airyscan processing. VP2 staining is shown in red, and STIM1 staining is shown in green; merged images are shown with areas of co-localisation in yellow. Scale bars were shown at bottom right of each picture.

To study whether vvIBDV infection activates STIM1 and CRAC channels, we performed Airyscan confocal microscopy assays to observe whether STIM1 shifts due to vvIBDV infection. Briefly, DT40 cells were infected by vvIBDV (MOI = 10) for 24 h. Following this, the fixed cells were incubated with mouse anti-VP2 antibody, rabbit anti-STIM1 antibody, and DAPI and analysed using Airyscan confocal microscopy. The results showed that in mock-infected cells, STIM1 was evenly distributed in the cytoplasm. However, vvIBDV-infection led to redistribution and oligomerization of STIM1 in the cell membrane of infected cells (Figure 7). These results suggest that vvIBDV infection might activates STIM1 proteins oligomerization and CRAC channels.

### 3.8. Inhibition of CRAC Channels Impairs vvIBDV Replication

**Figure 8:**
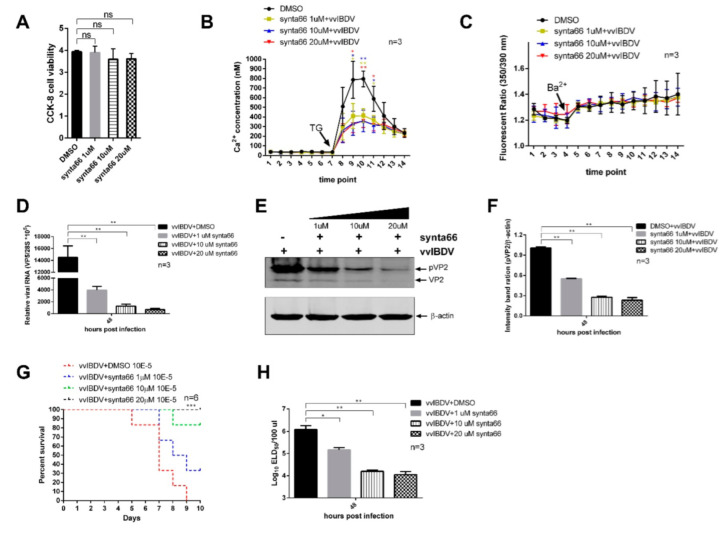
Inhibition of CRAC channels impairs vvIBDV replication. (**A**) CCK-8-based cell viability measurements demonstrated that Synta66 showed no significant cytotoxicity to DT40 cells compared with the DMSO group at concentrations of 1 μM, 10 μM, 20 μM for 48 h. Each value is normalized to cells treated with DMSO alone (*n* = 3). (**B**) 5MOI-infected DT40 cells were treated with Synta66 at indicated concentrations. Cells were harvested at 24 h p.i. and the intracellular Ca^2+^ levels were measured as described in Figure 3A. (**C**) DT40 cells were treated as described in (**B**). Cells were harvested at 24 h p.i. and the Ba^2+^ influx was measured as described in Figure 3B. Each unit represents 30 s. (**D**) DT40 cells were treated as described in (**B**). Cells were harvested at 48 h p.i. and RNA levels of VP5 were determined by RT-qPCR (*n* = 3). (**E**) 5MOI-infected DT40 cells were treated as described in B. Cells were harvested at 48 h p.i. and examined by WB with antibodies against pVP2 and VP2. Endogenous β-actin expression was used as an internal control. (**F**) Relative intensities of viral protein pVP2 in Synta66 or DMSO treated cells (*n* = 3). (**G**,**H**) 5MOI-infected DT40 cells were treated as described in B. At 48 h p.i., the percent survival of the chicken embryos was recorded in ((**E**), *n* = 6) and the infectious viral loads in the supernatants ((**F**), *n* = 6) were determined as described in Figure 4F. Mean ± SD were calculated based on three independent experiments. * *p* < 0.05; ** *p* < 0.01; *** *p* < 0.001.

To further confirm that CRAC channels were activated in vvIBDV infection, we used Synta66 (an inhibitor of Orai, which forms the core of CRAC channel) to evaluate the inhibition of CRAC channels on the Ca^2+^ levels and the replication of vvIBDV [30]. First, the cytotoxicity of Synta66 was determined. We found that Synta66 treated DT40 cells for 48 h at concentrations of 1 µM, 10 µM, and 20 µM had no significant difference with cells treated with comparable DMSO (vehicle control) (Figure 8A). Intracellular Ca^2+^ levels were checked in DT40 cells after infected with vvIBDV (MOI = 5), followed by Synta66 treatment for 24 h. The results showed that Synta66 treatment significantly decreased ER Ca^2+^ levels in a dose-dependent manner (Figure 8B). To rule out the cell membrane permeability as the cause of change in intracellular Ca^2+^ levels, we examined basal Ba^2+^ influx in Synta66 treated DT40 cells to study cell membrane permeability at 24 h p.i. As shown in (Figure 8C), the addition of Ba^2+^ to cells incubated in Ca^2+^-free HBSS buffer caused an insignificant increase in the fluorescence ratio in all the groups. These results suggest that the cell membrane permeability does not differ significantly between DMSO-treated cells and Synta66-treated cells (*p* > 0.05), indicating that change in cell membrane permeability was not responsible for the decrease in Ca^2+^ levels observed in Synta66 treated cells.

Upon analysing the relative expression of viral RNA by RT-qPCR, we found that Synta66-treatment (1 µM, 10 µM, and 20 µM) resulted in a respective 3-fold, 11-fold, and 21-fold decrease in the relative VP5 RNA transcript (Figure 8D). At the same time, a corresponding dose-dependent downregulation of viral proteins pVP2 and VP2 was observed in Synta66 treated cells (Figure 8E,F). Furthermore, the titre of progeny virus in supernatants of Synta66 treated vvIBDV-infected cells were tested with ELD_50_ assays. The results showed that Synta66 treatment (1 µM, 10 µM, and 20 µM) of infected cells resulted in 15-fold, 28-fold, and 31-fold decreases in extracellular viral yields at 48 h p.i. (Figure 8G,H). In conclusion, these results indicate that the inhibition of CRAC channels prevents extracellular Ca^2+^ entering the ER and leads to the decrease in ER Ca^2+^ levels, which impairs vvIBDV replication.

## 4. Discussion

The Ca^2+^ concentration in the blood serum is tightly regulated, and any abnormalities in serum Ca^2+^ level are associated with many dangerous diseases [31]. Our results showed vvIBDV infection resulted in significantly decreased Ca^2+^ levels in chickens’ serum. This result was consistent with previous research that Cosgrove found the most prominent finding in the chemical constituents of IBDV-infected chickens was the serum’s low Ca^2+^ content [6]. Such a severe imbalance of Ca^2+^ homeostasis would severely affect the normal physiological function of the host [32,33,34]. There have been many pieces of evidence that the dysregulation of Ca^2+^ is related to some major diseases in humans, such as cardiac disease, schizophrenia, bipolar disorder, and Alzheimer’s disease [35]. Serum Ca^2+^ levels were also found to be significantly correlated with dengue severity [36]. Hence, we wanted to understand the mechanism behind the significant decrease in serum Ca^2+^ levels in vvIBDV-infected chickens.

Cells live in extracellular fluid, of which serum is a part [37]. Thus, we analysed intracellular Ca^2+^ levels and found that the Ca^2+^ levels of the ER in vvIBDV-infected cells were significantly higher than in mock-infected cells. In the normal resting state of cells, the Ca^2+^ concentration inside and outside the cell maintains a dynamic balance [13,14,16]. However, several studies have shown that during the invasion of the host, many kinds of viruses can hijack and change the Ca^2+^ homeostasis of the host to fulfil their life cycle, such as coxsackievirus, human cytomegalovirus, and rotavirus [38,39,40]. Prior studies have also noted the importance of Ca^2+^ in the IBDV life cycle [41]. Taken together, vvIBDV infection results in a severe imbalance of Ca^2+^ homeostasis of the host, which might perform essential roles in the viral life cycle and pathogenesis.

The results of iTRAQ showed that the expression level of STIM1 significantly increased after vvIBDV infection. To date, several studies have suggested that many viral infections result in STIM1-mediated changes in Ca^2+^ levels. For example, constitutive STIM1 activation observed in rotavirus-infected cells reflects an effect of its non-structural protein 4 on ER Ca^2+^ permeability [26]. Ziying Han et al. provided evidence that host Ca^2+^ signalling, triggered by viral activation of STIM1 and Orai1, is among the key host mechanisms that orchestrate the later stages of filovirus and arenavirus assembly and budding [42]. Therefore, STIM1 was used as an ideal target protein to explore whether the changes in serum Ca^2+^ levels were caused by vvIBDV infection. Our results showed that vvIBDV infection induced upregulation of endogenous STIM1 protein levels. STIM1 overexpression has been reported to play important roles in the progression of many severe diseases, including several malignancies, such as pancreatic, breast, and cervical cancer [43,44,45]. To reveal the roles of upregulated expression of STIM1 in Ca^2+^ regulation and vvIBDV replication, we examined the effects of overexpression and knockdown of STIM1. Our results showed that overexpression of STIM1 increased Ca^2+^ levels in the ER, which improved vvIBDV replication, while knockdown of STIM1 decreased Ca^2+^ levels in the ER, which suppressed vvIBDV replication. In conclusion, we demonstrate that vvIBDV infection upregulates the expression of STIM1, and the elevated expression of STIM1 leads to increased Ca^2+^ levels in the ER, which facilitates vvIBDV replication. It suggests that increased Ca^2+^ levels in the ER are not related to Ca^2+^ store depletion but are related to the upregulated expression of STIM1 caused by vvIBDV infection. Studies have shown that except the decreased Ca^2+^ levels in ER can activate STIM1, STIM1 can also be activated by other factors, such as oxidant stress and temperature, which does not depend on Ca^2+^ store depletion [46,47]. Our results may provide new insight into STIM1 activation strategy.

Our results demonstrate that STIM1 plays a crucial role in vvIBDV–host cell interaction, but how this interaction influences Ca^2+^ levels in ER is unknown. Studies have shown that increased expression levels of STIM1 correlated with a gain in function of CRAC channel activity [48], while in STIM1 knockdown cells, significant suppression of Ca^2+^ influx was observed [49]. Thus, we sought to study how vvIBDV infection modulated ER Ca^2+^ levels in a STIM1-dependent manner and identify the viral proteins that interact with STIM1. CO-IP results indicated that VP2 and VP4 interacted with STIM1. It has been extensively studied that, in resting cells, STIM1 and Orai1 distribute independently in the ER and PM, respectively. However, activated STIM1 proteins oligomerize and accumulate at ER-PM junctions where it binds, traps, and opens CRAC channels for extracellular Ca^2+^ to enter the ER stores [23,29]. The appearance of STIM1 puncta can reliably indicate the activation of STIM1 [50,51]. STIM1 oligomerization is the critical transduction event, acting as a switch that triggers the self-organization and activation of STIM1-Orai1 clusters at ER-PM junctions, and whether or not the Ca^2+^ storage is emptied, subsequent steps will take place [24]. In our study, Airyscan confocal microscopy assays showed that STIM1 redistributed and oligomerized in the cell membrane of infected cells, indicating that STIM1 might be activated and accumulated at ER-PM junctions, leading to the activation of CRAC channels. To further confirm this conclusion, we employed the inhibitor of CRAC channels-Synta66 to verify if CRAC channels were activated in vvIBDV infection. Synta66 compound is one of the highest affinities CRAC channel inhibitor currently available and it can efficiently blocks store operated Ca^2+^ entry [30]. Our results showed that the inhibition of CRAC channels prevented extracellular Ca^2+^ entering the ER, resulting in the decrease in ER Ca^2+^ levels that impaired vvIBDV replication. This result can strongly demonstrate the previous conclusion that vvIBDV infection activated CRAC channels and allowed extracellular Ca^2+^ to enter the ER through CRAC channels. Furthermore, since CRAC channels are ubiquitously expressed, and like ion channels in general, they represent potentially pharmacologically accessible (cell surface) therapeutic targets with application in severe diseases with Ca^2+^ imbalance [42,51].

In conclusion, our study provides the first direct evidence that host Ca^2+^ signals triggered by the activation of STIM1 orchestrate the steps of vvIBDV replication. Viral proteins VP2 and VP4 interact with STIM1 and activates STIM1, leading to the opening of CRAC channels, which facilitates the entry of the extracellular Ca^2+^ into the ER. Indeed, this process may contribute to the decrease in serum Ca^2+^ level severe disturbance of Ca^2+^ homeostasis, which is worthy of further study in the future. Indeed, the pathogenesis of vvIBDV is quite complicated, and even metabolic disorder is the result of a combination of factors, such as difficulty feeding in late-infected chickens. Our study may provide new insights in the study of vvIBDV pathogenesis.

## Data Availability

Not applicable.
